# MiRiad Roles for MicroRNAs in Cardiac Development and Regeneration

**DOI:** 10.3390/cells3030724

**Published:** 2014-07-22

**Authors:** Ashley M. Fuller, Li Qian

**Affiliations:** 1 Department of Pathology and Laboratory Medicine, University of North Carolina, Chapel Hill, NC 27599, USA; E-Mail: amfuller@email.unc.edu; 2 McAllister Heart Institute, University of North Carolina, Chapel Hill, NC 27599, USA; 3 Lineberger Comprehensive Cancer Center, University of North Carolina, Chapel Hill, NC 27599, USA

**Keywords:** miRNA, cardiovascular development, cardiac regeneration, direct cell reprogramming, differentiation, myosin isoform switching

## Abstract

Cardiac development is an exquisitely regulated process that is sensitive to perturbations in transcriptional activity and gene dosage. Accordingly, congenital heart abnormalities are prevalent worldwide, and are estimated to occur in approximately 1% of live births. Recently, small non-coding RNAs, known as microRNAs, have emerged as critical components of the cardiogenic regulatory network, and have been shown to play numerous roles in the growth, differentiation, and morphogenesis of the developing heart. Moreover, the importance of miRNA function in cardiac development has facilitated the identification of prospective therapeutic targets for patients with congenital and acquired cardiac diseases. Here, we discuss findings attesting to the critical role of miRNAs in cardiogenesis and cardiac regeneration, and present evidence regarding the therapeutic potential of miRNAs for cardiovascular diseases.

## 1. Introduction

The survival of any metazoan during embryonic development and adult life depends on the continuous function of the heart. Accordingly, congenital heart abnormalities, which are estimated to affect approximately 1% of newborns [1,2,3], account for the greatest proportion of birth defect-related deaths in infants and young children in the United States [4]. Moreover, cardiovascular disease is the leading cause of mortality in both men and women worldwide [5]. Therefore, congenital and acquired cardiac diseases represent a major global health concern.

The mature heart is derived from a number of cell lineages, which differentiate coordinately into distinct regions with unique anatomic, physiological, and functional properties. Cardiac differentiation is regulated by an intricate, highly conserved network of transcription factors and co-regulators, and mutations in many of these factors have been implicated in the pathogenesis of both congenital and acquired cardiac abnormalities [1,6]. Briefly, cells within the first heart field, a group of myocardial progenitors originating from the anterior mesoderm, migrate from the primitive streak to the midline of the embryo where they form a crescent-shaped epithelium known as the cardiac crescent [1,7]. This structure then fuses to form a beating linear heart tube, which consists of cardiomyocytes and endothelial cells [8]. Subsequently, the heart tube undergoes rightward looping, a series of extensive morphological changes that contributes to the formation of the left ventricle and part of the atria [1,7]. Simultaneously, cardiac progenitors from the pharyngeal mesoderm, known as the second heart field, migrate to the anterior and posterior extremes of the heart tube where they give rise to the right ventricle, outflow tract, and portions of the atria. Importantly, proper looping and remodeling of the linear heart tube is essential for accurate positioning of the cardiac chambers and alignment of the valves [1].

Although the transcriptional modulation of cardiac development and disease has been an active area of investigation for many years, recent work has revealed a role for genes encoding short, non-protein-coding RNAs known as microRNAs (miRNAs) in many areas of cardiac biology. Due to the importance of miRNAs in the regulation of cardiac development and physiology, these studies may suggest novel prospective therapeutic targets for patients with congenital and acquired cardiac diseases [9]. In this review, we highlight recent and established findings that underscore the critical role of miRNAs in the regulation of cardiac development. We also present evidence regarding the therapeutic potential of miRNAs, specifically with respect to cardiac regeneration.

## 2. MiRNA Discovery, Biogenesis, and Mechanisms of Action

MicroRNAs are small, non-protein-coding RNAs, approximately 22 nucleotides in length, which repress gene expression by impairing mRNA stability or translation [10,11,12]. Victor Ambros, and colleagues Rosalind Lee and Rhonda Feinbaum, discovered the first miRNA, *lin-4*, in *Caenorhabditis elegans* (*C. elegans*) over two decades ago [13]. The *lin-4* gene was unusual because it resided within an intron of another, unrelated gene [13], and encoded two small RNA molecules with complementarity to repeated sequence elements within the 3' untranslated region (3'UTR) of the protein-coding *lin-14* transcript [13,14]. These findings were consistent with previous reports of *lin-4* functioning as a negative regulator of *lin-14* during early stages of *C. elegans* larval development [15]. Subsequently, Wightman *et al.* [14] demonstrated that the *lin-14* 3'UTR was necessary and sufficient for its *lin-4*-mediated post-transcriptional regulation, thereby supporting a model where the *lin-4* gene product could inhibit *lin-14* translation via direct binding to the 3'UTR of *lin-14* [13]. Since this initial discovery, it has been established that miRNAs are widely conserved across eukaryotes, and play fundamental roles in virtually all aspects of cell physiology. Currently, it is estimated that the human genome encodes over 800 miRNAs [16], which help to modulate the expression of nearly 30% of protein-coding genes [17].

MicroRNAs can be divided into several different groups based on their genomic organization and gene structure. Approximately 50% of miRNA genes are located within intergenic regions, and can be transcribed from their own promoters or as polycistronic clusters from a shared promoter [18,19]. The remaining annotated miRNAs are encoded within protein-coding genes, and are transcribed coincidentally with their host genes or from miRNA-specific promoters. The majority of these miRNAs are located within introns, although in rare cases, they may also overlap with protein-coding exons [18,20]. Most miRNA genes are transcribed in the nucleus by RNA polymerase II (Pol-II), and are regulated by Pol-II-dependent transcription factors in a cell type-specific manner [18,21]. However, a small subset of miRNAs, particularly those associated with Alu repeats, can also be transcribed by RNA polymerase III [22].

**Figure 1:**
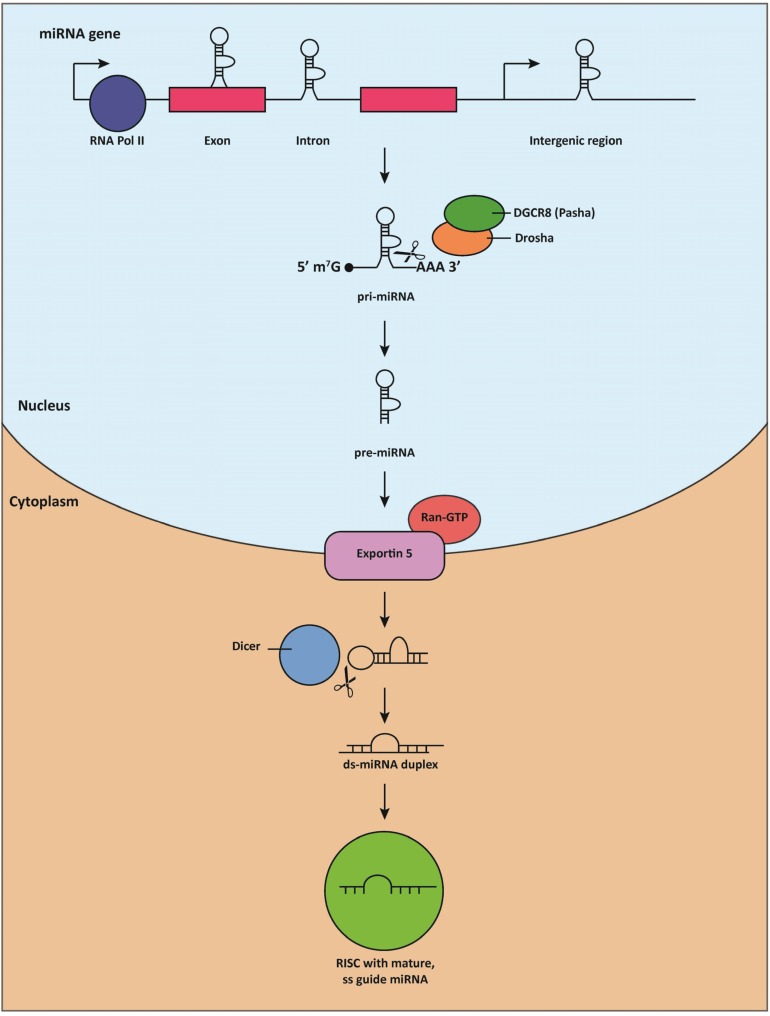
miRNA biogenesis.

Like other transcripts generated by Pol-II, miRNA transcripts contain a 5'7-methyl-guanosine cap and a 3' polyadenylation signal [23]. These transcripts, known as pri-miRNAs, are frequently several kilobases long and possess local hairpin secondary structure [24]. The first step of miRNA processing is the cleavage of the primary transcript into pre-miRNAs, stem-loop precursors that measure approximately 70 nucleotides in length (Figure 1). This process is mediated by the nuclear RNAse III-like enzyme, Drosha, and its cofactor, DGCR8 (DiGeorge syndrome critical region gene 8, or Pasha, Partner of Drosha, in invertebrates) [25,26,27,28]. Following Drosha-mediated cleavage, the pre-miRNA is exported to the cytoplasm by the RanGTP-dependent nuclear transport receptor, exportin 5 (EXP5) [29,30,31]. The terminal loop of the pre-miRNA is then cleaved by Dicer, a second highly conserved RNAse III-like enzyme [32], into a ~22-nucleotide duplex consisting of a “guide” strand and a “passenger” strand. The guide strand is loaded onto an Argonaut (Ago) protein as the mature miRNA [33,34,35], while the passenger strand is typically degraded [36]. Together, Ago, Dicer, and its cofactors constitute the RNA-induced silencing complex (RISC), which is responsible for guiding the mature miRNA to its target [37].

Given their short lengths, there is little sequence information available to confer specificity between miRNAs and their target mRNAs [38]. Accordingly, bioinformatics algorithms have predicted that each annotated miRNA possesses hundreds of target transcripts, although many of these interactions have not been functionally validated [39]. Typically, target recognition requires perfect base-pairing between the miRNA “seed sequence” (nucleotides 2–7 at the 5' end) and a complementary sequence within the 3'UTR of the mRNA target [40]. This interaction can facilitate gene silencing by a number of mechanisms, including translational inhibition, mRNA deadenylation, and endonucleolytic cleavage [reviewed in 38]. Unexpectedly, Vasudevan *et al.* recently reported that some miRNAs can also activate translation of their targets in a cell cycle-dependent fashion [41]. This observation has paramount implications for understanding the role of miRNAs in numerous biological processes, and suggests that miRNAs may possess a more diverse array of regulatory functions than initially appreciated.

MiRNAs are typically transcribed in the nucleus by RNA polymerase II (RNA Pol II), and can be encoded within protein-coding exons, the introns of protein-coding genes, or intergenic regions. Following transcription, the capped, poly-adenylated primary miRNA (pri-miRNA) is cleaved by the RNAse III-like enzyme, Drosha (Pasha in flies), and its partner, DGCR8, to generate a ~70 nucleotide-long pre-miRNA molecule that contains local stem-loop secondary structure. The pre-miRNA is then exported to the cytoplasm by the Ran-GTP-dependent nuclear transport receptor, exportin 5, where the terminal loop is cleaved by the RNAse III-like enzyme, Dicer. The resulting double-stranded ~22 nucleotide-long miRNA duplex consists of a “guide” and “passenger” strand; the “guide” strand is preferentially loaded into the RISC (RNA-induced silencing complex) as the mature miRNA, and ultimately guided to its target mRNA transcript.

## 3. MiRNAs Are Essential for Cardiac Development

### 3.1. Components the miRNA Biogenesis Pathway Are Required for Cardiac Development

One approach to evaluating the requirement for miRNAs during cardiac development has been to genetically manipulate Dicer, the enzyme responsible for processing pre-miRNAs into their mature forms. In vertebrates, this protein is encoded by a single gene, *Dicer1*. In mice, null mutations in *Dicer1* are lethal by approximately E7.5 (embryonic day 7.5), as a failure to process pre-miRNAs to maturity may result in the depletion of pluripotent stem cells (Table 1) [42]. Similarly, *Dicer1*-null zebrafish embryos exhibit generalized growth arrest, and typically do not survive beyond 13–14 days post-fertilization (d.p.f.) [43].

**Table 1:** miRNA mutations and corresponding cardiac phenotypes.

miRNA	Organism	Mutation	Phenotype	Citation
*Dicer1* (ribonuclease required for processing pre-miRNAs to maturity)	Zebrafish	Null	Growth arrest; death by 13–14 d.p.f	[43]
	Mouse	Null	Lethal (E7.5)	[42]
		CKO: Nkx2.5-Cre; Dicer^Fl/Fl^	Lethal (E12.5): myocardial abnormalities, pericardial edema	[44]
		CKO: Nkx2.5-Cre (3'UTR-IRES-Cre); Dicer^Fl/Fl^	Lethal (E13.5): VSD, DORV, reduced OFT apoptosis	[45]
		CKO: Wnt1-Cre; Dicer^Fl/Fl^	Loss of sympathetic neurons; morphological defects (type B interrupted aortic arch, VSD, DORV, retroesophageal right subclavian artery, ectopic carotids)	[46,47]
		CKO: αMHC-Cre; Dicer^Fl/Fl^	Dilative cardiomyopathy and heart failure; death by P4	[48]
		CKO: αMHC-MCM; Dicer^Fl/Fl^ (3-weeks old)	Spontaneous cardiac remodeling (mild RV inflammation, atrial enlargement); sudden death by 4 weeks of age	[49]
		CKO: αMHC-MCM; Dicer^Fl/Fl^ (adult)	Ventricular enlargement; cardiomyocyte hypertrophy and disarray; severe inflammation; interstitial ventricular fibrosis	[49]
*Dgcr8* (Cofactor required for cleavage of pri-miRNA hairpins)	Mouse	CKO: Wnt1-Cre; Dgcr8^Fl/Fl^	Persistent truncus arteriosis; VSD; interrupted aortic arch; cervical aortic arch; aberrant origin of right subclavian artery	[50]
		CKO: MCK-Cre; Dgcr8^Fl/Fl^	Reduced myocardial wall thickness; disrupted cardiac conduction; dilative cardiomyopathy; death by 2 months of age	[51]
*miR-1-1*	Mouse	Null: pGK-neomycin retained	Incompletely penetrant lethality (Sv129 background); ventricular dilation; conduction defects	[52]
*miR-1-2*	Mouse	Null: lacZ-pGK-neomycin retained	Incompletely penetrant lethality (E15.5-birth): VSD; cardiac dilation; atrial thrombosis; CM hyperplasia; conduction defects	[44]
*miR-1*	Drosophila	Null	Lethal (larval stages): Body wall collapse; striated muscle patterning defects	[53,54]
	Mouse	Null: neomycin-resistance cassettes retained	Lethal (P10): VSD, aortal misalignment; cardiac dilation; sarcomere disruption and retention of fetal sarcomere gene expression program	[52]
		Null: neomycin-resistance cassettes excised	Lethal (P17): Dilative cardiomyopathy, CM hyperplasia; retention of fetal sarcomere gene expression program	[55]
*miR-133a*	Mouse	Null	Incompletely penetrant lethality (P1): VSD; increased CM proliferation and ectopic smooth muscle gene expression	[56]
*miR-1/133*	Zebrafish	MO-*miR-1/133*	Disrupted sarcomeric actin organization (loss of I-bands)	[57]
	Mouse	Null	Lethal (E11.5): Impaired circulation, upregulation of smooth muscle gene expression	[58]
*miR-138*	Zebrafish	MO-*miR-138*	Retention of immature CM morphology; ectopic expression of genes restricted to AVC	[59]
*miR-218*	Zebrafish	MO-*miR-218*	Impaired migration of heart field progenitors; severe pericardial edema; morphological defects; ectopic expression of endothelial markers	[60,61]
*miR-92*	Zebrafish	*miR-92* mimic	Cardia bifida	[62]
		MO-*miR-92*	Left-right asymmetry defects	[62]
*miR-195*	Mouse	βMHC-miR-195 transgenic	Reduced CM proliferation; VSD; ventricular hypoplasia; dilative cardiomyopathy; premature death	[63]
*miR-17*	Mouse	*miR-17* transgenic	Reduced heart weight	[64]
*miR-17~92*	Mouse	Null	Perinatally lethal: VSD	[65]
		SM22α-Cre; *miR-17~92* transgenic	Cardiac hyperplasia and hypertrophy; sudden death by 2 months of age	[65]
*miR-17~92; miR-106b~25*	Mouse	Null	Embryonic lethal (E15): Ventricular hypoplasia, ASD, VSD, vascular congestion, edema	[66]
*miR-208a*	Mouse	Null	Sarcomere structural abnormalities, arrhythmias, ectopic expression of skeletal muscle genes	[67,68]

**Abbreviations**: ASD: atrial septal defect; AVC: atrioventricular canal; CM: cardiomyocyte; CKO: conditional knock-out; d.p.f.: days post-fertilization; DORV: double-outlet right ventricle; Fl/Fl: Homozygous floxed allele; MCM: Mer-Cre-Mer; MO: morpholino; OFT: outflow tract; RV: right ventricle; VSD: ventricular septal defect.

Due to the severity of global *Dicer1* loss-of-function mutations, tissue-specific deletion models have become essential to more completely understand the role of this enzyme in cardiac development. A conditional knockout of *Dicer1* using Cre recombinase under the control of endogenous Nkx2.5 (Nk2 homeobox 5) regulatory regions (Nkx2.5-Cre) ablated Dicer activity in cardiac progenitor cells beginning at E8.5 [44]. Embryos lacking *Dicer1* in the heart died by E12.5, and exhibited a poorly developed myocardium and pericardial edema. Interestingly, however, many key regulators of cardiac differentiation and patterning, such as Hand2 (heart and neural crest derivatives expressed), Tbx5 (T-box 5), and Mlc2v (myosin light chain 2v), were all expressed normally [44]. A second gene-targeted Nkx2.5-Cre line that made use of a different Cre allele (3'UTR-IRES-Cre) than the one previously employed by Zhao *et al.* [44] was subsequently used to reveal the requirement of Dicer for septation of the cardiac chambers and alignment of the outflow tract [45]. These embryos survived until approximately E13.75, slightly longer than the mutants generated in the preceding study. The extended survival of these mice was important because it permitted Tabin’s group to study the effects of *Dicer1* loss of function on morphological events that occurred too late during development to evaluate with the alternate Nkx2.5-Cre [45]. Taken together, although these two Nkx2.5-Cre lines exhibited slightly different spatiotemporal expression kinetics and manifested considerable phenotypic variation, the results of these studies indicate a requirement for cardiac-specific *Dicer1* expression in the development and morphogenesis of the heart.

Neural crest cells (NCCs) arise from the dorsal neural tube during early development, and differentiate to give rise to the peripheral nervous system, melanocytes, and craniofacial tissues. A subset of these cells, known as cardiac NCCs, are essential for various aspects of cardiac morphogenesis, including outflow tract septation, as well as development of the valves, the cardiac conduction system, and the pharyngeal arch arteries [46]. To investigate the effects of loss of Dicer activity in NCCs, Zehir *et al.* used a Wnt1-Cre line to conditionally delete *Dicer1* in this cell population. Although this mutation had no effect on NCC migration or target tissue colonization, NCC-specific deletion of *Dicer1* resulted in a loss of craniofacial bones, as well as enteric, sympathetic, and sensory neurons. Moreover, caspase-dependent apoptotic pathways were activated in the developing sensory ganglia and the sympathetic nervous system, indicating that Dicer activity is crucial for the survival of certain NCC-derived tissues [47]. A subsequent study with the same Wnt1-Cre line confirmed the requirement for Dicer in craniofacial development, but further demonstrated that Dicer loss of function did not affect the survival of tissues derived from cardiac NCCs. However, these mutant mice displayed many cardiovascular defects reminiscent of human congenital heart abnormalities, including type B interrupted aortic arch, double outlet right ventricle, ventricular septal defect (VSD), retroesophageal right subclavian artery, and ectopic placement of the carotid arteries [69]. Similar defects were observed in conditional, NCC-specific *Dgcr8* knockouts [50]. The effects of NCC-specific Dicer ablation were shown to be regulated in part by *miR-21* (*miRNA-21*) and *miR-181a*-mediated upregulation of Erk1/2 (extracellular signal-regulated kinase) signaling [69]. Importantly, however, aberrations in cardiac development have not been noted for either *miR-21* [70] or *miR-181a* knockout mice [71]. Therefore, studies that seek to characterize *miR-21/miR-181a* double knockout animals are required to further evaluate the role of these two miRNAs in NCC lineage specification. Alternatively, other miRNAs might be involved in NCC development that compensate for the functions of *miR-21/miR-181a* in the knockout mice.

In addition to its critical requirement during gestation, Dicer activity is also necessary for maintaining post-natal cardiac function and structural integrity. In contrast to the Nkx2.5-Cre lines, cardiomyocyte-specific Dicer ablation using an αMHC (α-myosin heavy chain)-Cre line was not embryonic lethal. However, dramatic reductions in the levels of mature miRNA in neonatal hearts were associated with features of dilative cardiomyopathy and heart failure, which resulted in death by post-natal day 4 (P4) [48]. To assess the requirement of Dicer activity in the hearts of older mice, Cre recombinase was expressed under the control of a tamoxifen-inducible αMHC promoter [49]. Cre-mediated excision of *Dicer1* in three week-old mouse hearts led to spontaneous cardiac remodeling, as evidenced by atrial enlargement and mild inflammation. These morphological changes resulted in a rapid loss of cardiac function, and high rates of sudden death by four weeks of age [49]. Spontaneous cardiac remodeling was also observed in adult mice harboring cardiomyocyte-specific *Dicer1* deletions. Unlike the younger mice, adult animals did not display premature sudden death. However, they exhibited more severe histopathological changes, including cardiomyocyte hypertrophy and disarray, extensive inflammation, and interstitial fibrosis [49]. A role for miRNA processing in the maintenance of cardiac function is further supported by muscle-specific deletion of *Dgcr8*. When expressed under the control of the MCK (myosin creatine kinase) promoter, Cre recombinase is activated in cardiac and skeletal muscle at birth, and declines to 40% of peak levels by P10 [51]. As with *Dicer1*-mutant mice, deletion of *Dgcr8* using MCK-Cre markedly reduced the thickness of the ventricular myocardium, and disrupted the cardiac conduction system. These severe perturbations in cardiac function ultimately progressed to dilated cardiomyopathy, and caused premature lethality by two months of age [51]. Together, these studies highlight the essential role of the miRNA biogenesis pathway in the maintenance of cardiac function and integrity during neonatal and adult life. The subsequent sections of this review will examine the functions of specific miRNAs and miRNA families in the regulation of cardiac development. Although recent findings have also uncovered a role for miRNAs in vasculogenesis and vascular biology, many comprehensive reviews on this topic have been published elsewhere [72,73,74,75], and will not be discussed here.

### 3.2. The mIR-1/133a Bicistronic Clusters Are Critical Regulators Cardiac Development

**Figure 2:**
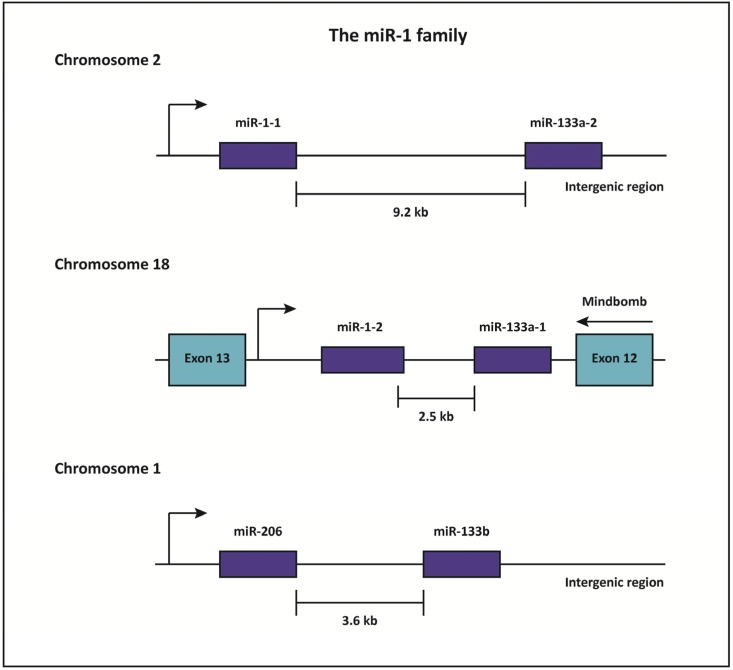
The *miR-1* family.

*miR-1* is an evolutionarily conserved miRNA that is highly enriched in cardiac and skeletal muscle [76,77,78]. Indeed, miRNA sequencing has revealed that *miR-1* is the most abundant miRNA in the mammalian heart, and accounts for approximately 40% of all miRNA expression in that tissue [51]. *miR-1* is transcribed as part of a bicistronic cluster together with *miR-133a*, a closely related miRNA that is also enriched in striated muscle [77]. The vertebrate genome contains two copies of the *miR-1/133a* locus, likely due to a gene duplication event [78]. Although these loci are located on different chromosomes (Figure 2), the mature miR-1 (*miR-1-1 and miR-1-2*) and miR-133a (*miR-133a-1 and miR-133a-2*) paralogs display complete sequence identity. An additional miRNA cluster consisting of *miR-206* and *miR-133b* is homologous to *miR-1*, but is expressed uniquely in skeletal muscle (Figure 2) [79].

Muscle-specific expression of the miR-1 and miR-133a family members is tightly controlled by a number of critical myogenic transcription factors, including SRF (serum response factor), Mef2 (myocyte enhancer factor-2), MyoD, myogenin, and Nkx2.5 [76,78,79,80]. In mice, mutation of predicted SRF binding sites within the *mIR-1-1* and *miR-1-2* enhancer regions disrupted *miR-1* expression in the heart, while cardiac-specific deletion of *Srf* ablated expression of the *miR-1* transcript [76]. In addition, Liu *et al.* [78] observed that expression of both *miR-1/133* bicistronic clusters was reduced in the hearts of *Mef2C*; *Mef2D* double knockout mice. Accordingly, they identified a muscle-specific, MEF2C-dependent enhancer located between the *miR-1-2* and *miR-133a-1* coding regions that directed *miR-1-2/133a-1* expression in the embryonic ventricular myocardium [78]. A similar enhancer was discovered between the *miR-1-1 and mIR-133a-2* genes, indicating that MEF2C controls the expression of both bicistronic miRNA clusters via analogous regulatory elements [78]. Similarly, in *Drosophila*, an SRF-like enhancer element is required for *miR-1* expression in cardiac progenitor cells [53], while Mef2 cooperates with the transcription factor Twist to regulate *miR-1* expression in late-stage embryos [54]. Moreover, a genetic screen in flies revealed that *miR-1* downregulates Cdc42 downstream of Tinman, the invertebrate ortholog of Nkx2.5; disruptions in this interaction severely impair adult cardiac physiology [80]. This interaction is also critical for maintaining cardiac contraction, electrical conduction, and rhythmicity in postnatal mice [80]. Taken together, the existence of highly conserved transcriptional networks directing *miR-1/133a* expression in the heart underscores the importance of these miRNAs in the regulation of cardiac development, as well as the maintenance of postnatal cardiac function.

*miR-1* and *miR-133a* are two closely related, striated muscle-enriched miRNAs that are transcribed together as members of a bicistronic cluster. The vertebrate genome contains two copies of the *miR-1/133a* locus on different chromosomes. The *miR-1-1/133a-2* cluster is located within an intergenic region of chromosome 2, while the *miR-1-2/133a-1* cluster is encoded within an intron of the protein-coding *Mindbomb* (*Mib*) gene. An additional intergenic miRNA cluster consisting of *miR-206* and *miR-133b* is located on chromosome 1. This cluster is homologous to *miR-1*, but is expressed exclusively in skeletal muscle.

Loss- and gain-of-function studies in *Drosophila* were the first to demonstrate the specific role of *miR-1* in the regulation of striated muscle development. Homozygous deletion of the single *Drosophila* *miR-1* ortholog (*dmiR-1*) was lethal, with 100% of flies dying in larval stages [53,54]. Mutant flies were profoundly lethargic as second instar larvae, and exhibited complete collapse of the body wall musculature [54]. In a similar study, down-regulation of critical sarcomere genes was observed in half of *dmiR-1* mutants, and severely affected embryos manifested defects in striated muscle patterning due to a failure of cardiac and skeletal muscle progenitors to terminally differentiate [53]. Overexpression of *dmiR-1* in the late mesoderm disrupted the formation of cardioblasts, indicating that an excess of *dmiR-1* may result in the premature differentiation of cardiac precursors, or divert cardiac progenitors towards an alternative cell fate [53]. Moreover, overexpression of *dmiR-1* in the developing *Drosophila* wing disks markedly reduced the protein levels of the Notch ligand, Delta, suggesting that the muscle patterning defects observed in these studies arose from deregulated Notch signaling [53].

Similar studies have also uncovered a role for *miR-1* in the regulation of cardiac development in various other model organisms. In mice, overexpression of *miR-1* in cardiomyocytes arrested embryonic development by E13.5 secondary to heart failure and ventricular abnormalities [76]. In transgenic embryos, a significant reduction in the number of mitotic cardiomyocytes was observed relative to controls. This phenotype was consistent with premature cardiac progenitor differentiation, early withdrawal of cardiomyocytes from the cell cycle, and an insufficient expansion of ventricular cardiomyocytes [76]. Hand2, a cardiogenic transcription factor essential for cardiac differentiation, was further implicated as an *in vivo* target of *miR-1*. This finding supported a model in which *miR-1* regulates Hand2 temporally and spatially during cardiac development [76].

Genetic knockout of *miR-1-2* in mice provided the first evidence regarding the essential role of an individual *miR-1* family member in cardiogenesis. Targeted *miR-1-2* deletion on a pure Sv129 background resulted in incompletely penetrant lethality from E15.5 to birth, secondary to VSD [44,52]. The remaining homozygotes that survived to adulthood exhibited a spectrum of cardiac defects, including rapid cardiac dilation, atrial thrombosis, and various cardiac conduction abnormalities. Consistent with previous reports implicating *miR-1* in the control of embryonic cardiomyocyte expansion [76], *miR-1-2* null mice that survived to adulthood exhibited a 20% increase in the number of cardiomyocytes relative to wild-type controls. Remarkably, a significant increase in the number of cardiomyocytes undergoing nuclear division was also detected in mutant adults, a phenomenon not observed in wild type animals of the same age; concomitant increases in cardiomyocyte apoptosis were not detected [44]. Similarly, *miR-1-1*-null animals exhibited a number of cardiac defects, including conduction abnormalities and cardiac dilation, as well as incompletely penetrant lethality on a pure Sv129 background [52]. In contrast to these findings, however, a recent study found that targeted deletion of *miR-1-1* or *miR-1-2* caused neither embryonic lethality nor septal defects in Sv129 mice [55]. These phenotypic discrepancies may be attributed to residual pGK-neomycin and lacZ-pGK-neomyocin cassette expression in the previously reported *miR-1-1* [52] and *miR-1-2* [44] knockout mice, respectively [55].

When *miR-1-1* and *miR-1-2* single knockouts were intercrossed to generate mice completely lacking *miR-1* (*miR-1-1^−/−^; miR-1-2^−/−^*), approximately 25% of animals died shortly after birth, likely resulting from VSD and misalignment of the aorta [52]. However, these congenital cardiac defects were not observed in *miR-1-1/1-2* double knockouts in which positive selection cassettes had been excised [55]. No *miR-1-1*/*1-2* double knockout animals survived to adulthood due to cardiac chamber dilation and severely impaired cardiac function [52,55]. *miR-1*-null cardiomyocytes also exhibited extensive sarcomeric disarray, as well as a marked upregulation of smooth muscle-specific and fetal sarcomere-associated genes such as *Telokin*, *Myocardin*, *Acta1*, *Acta2*, and βMHC/*Myh7* [52,55]. Although the mechanisms by which *miR-1* regulates these critical cardiac genes are only beginning to be elucidated, the induction of a fetal cardiac genetic program in *miR-1*-null animals was proposed to occur via the upregulation of e*strogen-related receptor β* (*Errβ*), an important regulator of glycolysis and glycogenesis [55].

Similar to the *miR-1-1* and *miR-1-2* single knockout mice described by Wei *et al.* [55], animals lacking either *miR-133a-1* or *miR-133a-2* in which the neomycin selection cassette had been excised showed no apparent defects in cardiac physiology, and responded to cardiac pressure overload in a manner indistinguishable from wild-type controls [56]. However, 50% of *miR-133a-1*; *miR-133a-2* double knockouts died between P0 and P1 from severe VSDs, and only 25% of compound homozygotes survived to adulthood [56]. Consistent with severe defects in cardiac contraction and function, adult double knockout mice exhibited increased cardiomyocyte proliferation, enhanced expression of cell cycle-related genes, and ectopic expression of smooth muscle genes in the heart [56]. Moreover, cardiac-specific overexpression of *miR-133a* caused a spectrum of abnormalities in the developing heart, including diminished cardiomyocyte proliferation within the ventricular myocardium, and death by E15.5 secondary to cardiac failure. Many of these phenotypes were attributed to the upregulation of the genes encoding SRF and cyclin D2, which are both normally targeted for repression by *miR-133a* [56]. Taken together, these data indicate that *miR-133a* plays a critical role in the regulation of contractile gene expression and cardiomyocyte proliferation during embryogenesis, and suggest a possible role for *miR-133a* in mediating cardiomyocyte cell cycle withdrawal in early postnatal life.

Due to the fact that *miR-1* and *miR-133a* possess different seed sequences, it is reasonable to assume that they regulate a separate set of target mRNAs and have distinct biological functions. Remarkably, however, there exists a significant functional overlap between the members of these two miRNA families with regard to their respective roles in cardiac development. For example, recent affinity purification data demonstrated that Hand2, a well-characterized target of *miR-1*, is also regulated by *miR-133a in vitro* and *in vivo* [81]. This study was important because it empirically established Hand2 as a functional *miR-133a* target, despite the inability of several miRNA prediction algorithms to identify this interaction [81]. Similarly, a study using zebrafish depleted of muscle-specific miRNAs showed that approximately 68% of upregulated transcripts possessed canonical 6mer, 7mer, or 8mer binding sites for *miR-1* or *miR-133a* [57]. Moreover, concomitant morpholino-mediated knockdown of *miR-1* and *miR-133a* manifested more severe disruptions in sarcomeric actin organization than knockdown of either miRNA alone, suggesting that *miR-1* and *miR-133a* cooperatively regulate actin polymerization and sarcomere assembly [57]. Finally, in mice, genetic deletion of both *miR-1*/*miR-133a* clusters resulted in death by E11.5 secondary to severe impairments in blood circulation and cardiac function [58]. Consistent with reports of increased smooth muscle gene expression in *miR-1-1/miR-1-2* [52] and *miR-133a-1/133a-2* [56] double knockout hearts, transcriptional profiling of *miR-1/133a* compound knockouts revealed an upregulation of *myocardin*, which is a major regulator of smooth muscle gene expression [58]. Accordingly, although early stages of heart development such as linear heart tube formation proceeded normally in *miR-1*/*133a* mutant embryos, double knockout hearts consistently displayed a gene expression pattern characteristic of immature cardiomyocytes, in that they expressed both cardiac- and smooth-muscle specific transcripts [58]. Taken together, these findings indicate a novel role for the *miR-1/133a* bicistronic clusters in the transition of immature cardiomyocytes to a more mature phenotype, and illustrate the complexity of miRNA-mediated spatial and temporal modulation of gene expression.

### 3.3. miR-138 and miR-218 Control Cardiac Patterning

The acquisition of cardiac cell identity and the establishment of domain-specific function are dependent upon a complex network of transcriptional and post-transcriptional regulatory factors [82]. In vertebrates, heart development culminates in the formation of septated atrial and ventricular chambers, which exhibit unique gene expression patterns, and thus, possess distinct morphological, electrical, and functional properties. These chambers are separated by the atrioventricular canal (AVC), a discrete structure that gives rise to the cardiac valves and ultimately ensures the unidirectional flow of blood through the heart [83]. Studies using zebrafish have identified distinct gene expression profiles that distinguish the AVC from the surrounding cardiac chambers [84], and have uncovered a role for miRNAs in the regulation of associated cardiac patterning events. Specifically, when injected with a morpholino targeting *miR-138*, zebrafish embryonic ventricular cardiomyocytes failed to elongate, and instead, remained in a rounded, immature state reminiscent of cardiomyocytes within the AVC [59]. Moreover, genes normally confined to the AVC, such as *notch1b* and *espg2*, were ectopically expressed in the ventricle, indicating that *miR-138* restricts the valve-forming region by repressing AVC-specific genes in maturing ventricular cardiomyocytes. These effects were attributed in part to *miR-138*-mediated downregulation of *aldh1a2*, which is required for the synthesis of retinoic acid, as well as the upregulation of *espg2* in the AVC [59]. Although these findings have important implications for understanding the regulation of cardiac patterning in zebrafish, future studies are required to assess the requirement of *miR-138* for the specification of cardiac domains in mammals.

Further support attesting to the importance of miRNAs in the regulation of cardiac morphogenesis and patterning has originated from studies of the *miR-218* family in zebrafish. In mammals, the *miR-218* family consists of two highly conserved members. *miR-218a-1* and *miR-218-a-2* are located within introns of the *slit3* and *slit2* genes, respectively; in fish, a third family member, *miR-218b*, is encoded intergenically. Although better known as an emerging cancer biomarker [85,86,87] and for its role in directing axon guidance during nervous system development [88,89], *miR-218*-mediated modulation of Slit-Robo (Roundabout) signaling is also critical for the patterning of the developing heart [60] and vasculature [90]. Injection of one- and two-cell stage zebrafish embryos with *miR-218*-targeting morpholinos impaired the migration of heart field progenitor cells to the midline during heart tube formation, and caused severe pericardial edema and cardiac morphological defects 48 hours post-fertilization (h.p.f.) [60]. Partial knockdown of *robo1* rescued the morphant phenotype, implying that *miR-218* regulates cardiac progenitor migration via direct *robo1* repression [60]. However, using the same morpholinos as Fish *et al.* [60], Chiavacci *et al.* did not observe any cardiac cell migratory or morphological defects at 48 h.p.f., which was attributed to the extremely low levels of endogenous *miR-218* expression at early developmental stages [61]. Accordingly, the authors reported abnormal cardiac looping, chamber malformations, and ectopic expression of *tie2*, a marker restricted to endothelial and hematopoietic cells, following *miR-218* overexpression. Moreover, it was shown that *miR-218* and *tbx5* expression correlate *in vitro* and *in vivo*, and that many of the cardiac defects associated with *tbx5* overexpression could be rescued by *miR-218* downregulation [61]. Due to the fact that *Tbx5* haploinsufficiency underlies the pathogenesis of Holt-Oram syndrome in humans, a congenital disease characterized by cardiac septation defects [91], further studies are required to assess the function of the *miR-218* – *Tbx5* regulatory circuit in vertebrate heart morphogenesis and patterning.

### 3.4. The miR-15 Family Negatively Regulates Cell Proliferation and Induces Embryonic Cardiomyocyte Mitotic Arrest

Cardiomyocytes undergo extensive proliferation during mammalian embryogenesis, yet withdraw from the cell cycle in early post-natal life [92]. This developmental switch is characterized by a transition from hyperplastic to hypertrophic cardiac growth, and is marked by the formation of binucleated cardiomyocytes, the maturation of intercalated discs, and an increase in myofibril density [93]. In order to identify miRNAs associated with this transition, Porrello *et al.* examined the developmental expression pattern of miRNAs in rodent ventricular cardiomyocytes as they approached cell cycle arrest [63]. These profiling experiments revealed a dramatic upregulation of multiple miR-15 family members, which corresponded with the onset of cardiomyocyte binucleation. Overexpression of the most highly upregulated miR-15 family member, *miR-195*, in the embryonic heart significantly reduced cardiomyocyte proliferation, and led to VSDs and ventricular hypoplasia in approximately 25% of neonatal mice. Moreover, *miR-195* transgenic animals that survived to adulthood died prematurely due to the development of a slow-onset dilative cardiomyopathy [63]. Taken together, these data indicate that the miR-15 family plays an important role in the mediation of cardiomyocyte senescence in early post-natal life.

The propensity of miR-15 family members to negatively regulate cell proliferation has been attributed to their *in vitro* ability to directly target components of the core cell cycle machinery, such as *cyclin D1*, *cyclin D3*, and *cyclin E* [94,95]. In addition, experiments using a variety of cancer cell lines have revealed that the *miR-15* and *miR-16* clusters are direct targets of E2F, a critical transcription factor that mediates the transition from G1- to S-phase [95]. Interestingly, E2F is a downstream mediator of the tumor suppressor protein pRb, which was recently shown to maintain adult cardiomyocytes in a post-mitotic state by directing heterochromatin formation at the promoters of cell cycle-regulated genes [96]. Analysis of neonatal *miR-195* transgenic mouse hearts using next-generation massively-parallel sequencing of RISC-associated transcripts (RISC-seq) further demonstrated that the miR-15 family targets a number of cell cycle-related genes *in vivo* [63]. One of these genes, *Chek1* (checkpoint kinase 1), plays a number of roles during DNA repair and mitosis, including the prevention of genomic instability, and the coordination of G2/M progression [63]. Although this experiment provides some insight into the mechanisms by which the miR-15 family may regulate neonatal cardiomyocyte mitotic arrest, the function of *Chek1* in cardiac development remains poorly understood, and therefore warrants further study.

### 3.5. Emerging, Multifaceted Roles for the miR-17~92 Cluster in Cardiac Development

In humans, the *miR-17~92* cluster, also known as OncomiR-1, is a highly conserved, polycistronic transcript that encodes six miRNAs (*miR-17*, *miR-18a*, *miR-19a*, *miR-19b-1*, *miR-20a*, and *miR-92-1*) in four families. In addition, ancient gene duplication events have given rise to two mammalian *miR-17~92* paralogs, including the *miR-106b~25* and *miR-106a~363* clusters. The *miR-17~92* and *miR-106b~25* clusters are highly expressed in many tissues. However, *miR-106a~363* expression has not been detected in any cell type studied, indicating that this transcript represents a non-functional pseudogene, or possesses an extremely specialized function [97].

Several studies have revealed important roles for the *miR-17~92* cluster and its paralogs in cardiac development. Although mice homozygous null for either the *miR-106b~25* or *miR-106a~363* cluster did not display an overt phenotype, animals lacking the *miR-17~92* locus died within minutes after birth due to VSDs and lung hypoplasia [66]. Moreover, compound deletion of *miR-17~92* and *miR-106b~25* was embryonic lethal, and resulted in a range of severe cardiovascular abnormalities including ventricular hypoplasia, atrial and ventricular septal defects, vascular congestion, and edema. The effects of *miR-17~92* deletion were attributed in part to upregulation of pro-apoptotic proteins such as *Bim*, which was shown to be a direct target of this miRNA family [66]. Although further studies are required to more thoroughly assess the role of *Bim* deregulation on the cardiac phenotypes caused by *miR-17~92* deletion, these results illustrate the essential and cooperative functions of *miR-17~92* and its paralogs in cardiac development.

The *miR-17-92* cluster has also been shown to influence the differentiation of second heart field progenitors, which contribute to the development of the right ventricle and outflow tract. During embryogenesis, cells of the second heart field are distinguished by the expression of the critical cardiogenic transcription factors *Isl1* (ISL LIM homeobox 1) and *Tbx1* (T-box 1) [98], which are transiently expressed prior to the initiation of cardiac progenitor cell differentiation [99,100]. In mice, compound deletion of *Bmp2/4* (bone morphogenic protein) resulted in defective silencing of these cardiac progenitor genes, as well as a downregulation of cardiac differentiation markers [101]. This result was attributed to direct BMP-mediated regulation of *pri-miR-17~92* expression, suggesting that BMP signaling is critical for the modulation of cardiac progenitor gene dosage [101].

Additional studies in zebrafish have revealed that *miR-17~92* is an important regulator of cardiac morphogenesis and patterning. Specifically, injection of *miR-92* mimics into the one-stage embryos was associated with reduced endoderm formation and cardia bifida, while morpholino-mediated *miR-92* depletion resulted in left-right cardiac asymmetry defects [62]. Moreover, members of the *miR-17~92* cluster have also been implicated in the regulation of organ size. Mice globally overexpressing a *miR-17* transgene were smaller than their control littermates, and exhibited significant reductions in heart, liver, and spleen weight, likely due to *miR-17*-mediated repression of fibronectin expression [64]. However, overexpression of the entire *miR-17~92* cluster in developing murine cardiomyocytes resulted in cardiac hyperplasia and hypertrophy, and caused sudden death by approximately two months of age [65]. The discrepancies between these two studies [64,65] suggest that members of the *miR-17~92* cluster possess distinct functions, and illustrate the need for further studies to identify the roles of each individual miRNA in the regulation of cardiac development and function.

### 3.6. The myomiR Mediates Myosin Heavy Chain Isoform Switching during Fetal and Adult Stages and Helps Maintain Proper Cardiac Electrophysiology

Myosin is a major molecular component of cardiac muscle, and is the protein responsible for the generation of contractile force in that tissue. Cardiac contraction is primarily dependent upon the expression of two *MHC* (myosin heavy chain) genes, α and β, which are regulated in an antithetical manner in response to developmental and pathological signals [102]. In rodents, βMHC/*Myh7*, a slow adenosine triphosphatase (ATPase), is the predominant myosin isoform expressed in the embryonic heart, whereas αMHC/*Myh6*, a relatively faster ATPase, is highly upregulated during the early post-natal period [103,104]. By contrast, in humans and other large mammals, the expression of the βMHC isoform is retained during adulthood [102], suggesting that species-specific regulatory mechanisms underlie the control of myosin expression.

A recent series of findings has demonstrated that myosin isoform expression is regulated in part by a vertebrate-specific family of miRNAs known as the “myomiR.” This miRNA family consists of *miR-208a*, *miR-208b*, and *miR-499*, which are encoded within the introns of *Myh6*, *Myh7*, and the closely related *Myh7b*, respectively. Interestingly, these miRNAs are expressed concomitantly with their host genes: In mice, *miR-208b* and *miR-499* are most highly expressed during embryonic development, while *miR-208a* is upregulated shortly after birth [67,68,105]. Moreover, the members of the myomiR family possess closely overlapping “seed” sequences, indicating that they likely regulate a similar array of target transcripts.

Genetic loss-of-function studies in mice have demonstrated that individual myomiRs are not explicitly required for cardiac development. Animals null for *miR-208a*, *miR-208b*, or *miR-499* are obtained at Mendelian ratios, and exhibit no overt developmental abnormalities or cardiac contractility defects prior to weaning [67,68,105]. However, the high degree of sequence similarity among these three miRNAs may confer functional redundancy, and further experiments employing compound knockout animals are required to fully assess the role of the myomiR trio in cardiogenesis. Interestingly, however, one study reported a potential role for *miR-208a* in the maintenance of cardiac contraction during adult life. Specifically, *miR-208a*-deficient animals experienced progressive declines in cardiac function beginning at approximately two months of age, and manifested severe abnormalities in sarcomere structure and ectopic expression of skeletal muscle-specific genes at six months of age [67]. Consistent with defects in cardiac contraction, electrocardiography (ECG) readings from four month-old *miR-208a* knockout mice lacked P-waves [68]. Given that P-waves normally represent atrial depolarization and contraction, the loss of P-waves in *miR-208a* knockout mice is suggestive of atrial fibrillation [68]. Likewise, cardiac-specific overexpression of *miR-208a* resulted in an elongation of the PR intervals in transgenic mice, indicative of what is clinically considered first-degree AV (atrioventricular) block. These electrophysiological abnormalities were attributed to interactions between *miR-208a* and the gap junction protein Cx40 (connexin 40), and transcription factors normally expressed in the cardiac conduction system such as GATA4 (GATA binding protein 4) and Hop (homeodomain-only protein) [68]. However, contrary to the findings of van Rooij *et al.* [67] and Callis *et al.* [68], another study demonstrated that systemic administration of *miR-208a* antimiRs in adult mice did not induce changes in cardiac conduction properties [106]. Therefore, further experiments are required to more clearly define the role of *miR-208a* and the remaining members of the myomiR family in the maintenance of proper cardiac electrophysiology.

Although loss of *miR-208a* manifests only minor defects with regard to cardiac development and function, this miRNA plays a crucial role in mediating certain aspects of the cardiac stress response. Reactivation of the fetal cardiac genetic program, including myosin isoform switching, is a characteristic of the stressed, hypertrophic, or failing post-natal heart [107]. In response to TAB (thoracic aortic banding), a procedure that reliably induces hypertrophic growth in the hearts of wild-type mice by increasing cardiac afterload, *miR-208a*-deficient animals displayed minimal signs of cardiac hypertrophy or fibrosis [67]. Although the expression of cardiac stress markers, such as atrial natriuretic factor (ANF) and b-type natriuretic peptide (BNP), was unaffected in the hearts of *miR-208a* knockout mice, these animals were remarkably unable to upregulate βMHC [67,68]. Similarly, cardiomyocyte-specific overexpression of *miR-208a* was sufficient to induce βMHC expression in the heart, even in the absence of overt cardiac pathology. These effects were proposed to result in part from *miR-208*-mediated repression of THRAP1 (thyroid hormone receptor-associated protein 1), a transcriptional co-regulator of thyroid hormone signaling [67,68], and myostatin, a suppressor of skeletal muscle hypertrophy [68]. Due to the fact that loss of *miR-208a* during embryogenesis has no effect on βMHC protein levels [67,68,105], it will be interesting to determine if distinct regulatory mechanisms underlie βMHC expression during development and situations of cardiac stress.

## 4. MiRNAs as Emerging Therapeutic Targets for Cardiac Regeneration

### 4.1. MiRNAs and Proliferation Endogenous Cardiomyocytes

Historically, the heart was considered a terminally differentiated organ incapable of proliferation or regeneration. However, this dogma has been challenged by recent reports of limited yet significant cardiomyocyte turnover in adults during aging [108,109], as well as following myocardial infarction [109]. Nevertheless, the intrinsic regenerative capacity of the adult mammalian myocardium is not sufficient to compensate for the loss of cardiac function post-injury, and ischemic cardiac disease remains the leading cause of adult morbidity and mortality worldwide. In contrast to the negligible innate regenerative ability of the adult heart, Porrello *et al.* recently demonstrated that the hearts of neonatal mice possess a robust capacity for regeneration during the first week of life in response to resection of the ventricular apex [92] and myocardial infarction [110]. Interestingly, this response is lost in seven day-old mice, a time point corresponding to the withdrawal of cardiomyocytes from the cell cycle [92,110].

The proliferation of existing cardiomyocytes has been identified as the primary mechanism underlying cardiac regeneration in neonatal mice [92,110] and lower vertebrates such as zebrafish [111] and amphibians [112]. Due to the important role of the *miR-15* family in the modulation of cardiomyocyte proliferation during cardiogenesis, the functions of these miRNAs have also been investigated in the setting of cardiac regeneration. Neonatal transgenic animals overexpressing the *miR-15* family member *miR-195* in cardiomyocytes fail to mount a regenerative response to myocardial infarction, and exhibit features characteristic of adult cardiac remodeling including inflammation, cardiomyocyte hypertrophy, and fibrosis [110]. Similarly, systemic antimiR-mediated inhibition of the *miR-15* family from birth to adulthood induced cardiomyocyte proliferation in infarcted adult hearts, and elicited improvements in left ventricular systolic function [110]. Although these data indicate that members of the *miR-15* family may be valid targets for patients with ischemic cardiac disease, systemic antimiR administration may elicit off-target effects. Therefore, further studies that evaluate the safety and efficacy of cardiomyocyte-specific *miR-15* inhibition are required before this approach is attempted in clinical trials.

The observation that the *miR-17~92* cluster is required for the proliferation of cardiomyocytes in embryonic, postnatal, and adult hearts [113] suggests that these miRNAs may be attractive therapeutic targets for patients with cardiac disease. To this end, transgenic embryos overexpressing *miR-17~92* in the heart displayed enhanced cardiomyocyte proliferation relative to control littermates. Moreover, overexpression of the *miR-17~92* cluster induced adult cardiomyocyte proliferation in healthy animals, as well as in response to myocardial infarction [113]. These effects were attributed to *miR-17~92*-mediated inhibition of the tumor-suppressor protein PTEN (phosphatase and tensin homolog) [113]. However, due to the role of this miRNA cluster in tissue growth and tumorigenesis, further studies evaluating the safety of cardiomyocyte-specific *miR-17~92* overexpression are required before these data can be applied to human patients.

Additional support for the role of miRNAs in cardiac regeneration has originated from a functional screen seeking to identify mature miRNAs capable of inducing cardiomyocyte proliferation [114]. Forty miRNAs were shown to enhance DNA synthesis in neonatal rodent cardiomyocytes, while two miRNAs, *miR-199a* and *miR-590*, were capable of inducing cell-cycle re-entry in adult rat cardiomyocytes *in vitro*. Moreover, AAV9 (adeno-associated virus serotype 9)-mediated *in vivo* delivery of *miR-199a* or *miR-590* elicited a significant regenerative response in adult animals following myocardial infarction [114]. This study provides compelling evidence for the utility of miRNAs to *in vivo* cardiac regeneration, and it will be interesting to evaluate the roles of endogenous *miR-199a* and *miR-590* during cardiac development.

### 4.2. MiRNAs and Cell-Based Strategies for Cardiac Regeneration

The field of stem cell biology is rapidly expanding and holds great potential for cardiac regeneration. Indeed, cardiomyocytes derived from embryonic stem cells (ESCs) and resident cardiac progenitor cells (CPCs) have imparted considerable functional improvements in animal models of ischemic heart disease [115]. However, clinical trials of these cell-based therapies have only reported modest, transient results, and the benefits of stem cells for cardiac regeneration in human patients with ischemic heart disease have not been conclusively established. Further studies are required to improve the safety of cell transplantation, and to overcome challenges such as poor engraftment rates, immunomodulation of engrafted cells, and patient heterogeneity [116]. One approach to improving the feasibility of these therapies for heart disease has been to incorporate miRNAs involved in the regulation of stem cell potency and self-renewal, as well as cardiomyocyte lineage commitment and differentiation. Although numerous studies attest to the potential of miRNAs to enhance cell transplantation therapies for cardiac disease, these findings have been reviewed extensively elsewhere [117,118,119,120], and will not be a focus of this review.

An alternative strategy to the use of ESCs and CPCs for cardiac regeneration is lineage reprogramming, an approach whereby one cell type is effectively converted into another. Historically, it was believed that the differentiation of somatic cells represented a permanent restriction of cell plasticity [121]. However, this dogma was overturned by somatic cell nuclear transfer experiments, which demonstrated that the nucleus of a somatic cell could revert to a pluripotent state and support the development of an entire embryo when transplanted into an enucleated oocyte [122,123]. An explosion in the cell reprogramming field occurred following Shinya Yamanaka’s landmark discovery that embryonic and adult murine fibroblasts could be induced to pluripotency by the introduction of the transcription factors *Oct3/4*, *Sox2*, *c-Myc*, and *Klf4* [124]. Interestingly, expression of the *miR302/367* cluster in the presence of the HDAC inhibitor valproic acid was shown to revert mouse and human fibroblasts to pluripotency more efficiently than the original reprogramming factors [125]. These induced pluripotent stem cells (iPSCs), which can be differentiated into virtually any somatic cell type following the addition of lineage-specific signaling molecules, have revolutionized the field of regenerative medicine. They represent a source of patient-specific stem cells amenable to disease modeling, drug discovery, and the development of personalized therapies [126].

In attempt to address concerns regarding the tumorigenic potential of iPSCs and the immaturity of iPSC-derived cardiomyocytes, several groups have adopted a direct reprogramming strategy, in which one somatic cell type is transdifferentiated into another. The mammalian heart is a rich reservoir of cardiac fibroblasts, which function as a structural scaffold for cardiomyocytes during embryogenesis [127], and contribute to extracellular matrix homeostasis in adults [128]. Cardiac fibroblasts also mediate the response to ischemic cardiac injury by proliferating and migrating to the infarct area where they orchestrate tissue remodeling, collagen deposition, and scar formation [129]. Ultimately, the loss of contractility within the infarcted region contributes to ventricular dysfunction, mechanical cardiac overload, and the development of chronic heart failure [130,131]. Due to their relative abundance in the heart and their fundamental role in the response to cardiac injury, Ieda *et al.* speculated that cardiac fibroblasts could represent a source of cardiomyocytes for regenerative therapies [132]. They systematically screened a group of key cardiogenesis regulators in order to identify factors capable of directly reprogramming cardiac fibroblasts into cardiomyocytes; ectopic expression of the transcription factors *Gata4*, *Mef2C*, and *Tbx5* (*GMT*) was necessary and sufficient to generate cardiomyocyte-like cells *in vitro* [132]. Although the generation of functional, beating cells occurred at a very low frequency, Qian *et al.* [133] and Song *et al.* [134] subsequently showed that *GMT*-mediated reprogramming could also occur *in vivo*, and impart significant functional improvements in murine models of myocardial infarction.

Despite the apparent promise of lineage reprogramming to cardiac regeneration, many of these approaches rely on the use of virally encoded transcription factors. This feature of current reprogramming technology raises concerns due to the possibility of insertional mutagenesis [135]. In order to improve the safety of reprogrammed cells, alternative reprogramming strategies that circumvent the use of integrating viral vectors are being developed. For example, it was recently shown that when transiently transfected with *miR-1*, *-133*, *-208*, and *-499*, cardiac fibroblasts could acquire a gene and protein expression profile consistent with that of cardiomyocytes [136]. This study also provided proof-of-principle evidence that this miRNA cocktail could mediate direct cardiac reprogramming in a mouse model of cardiac injury [136]. However, because this latter experiment employed lentivirally-encoded miRNAs, it is not clear whether *in vivo* reprogramming can be accomplished with transiently transfected cardiac fibroblasts. Moreover, the authors did not indicate whether the induced cells were capable of improving cardiac function after injury. Therefore, additional functional data are required to fully assess the utility of miRNAs to *in vivo* direct cardiac reprogramming.

## 5. Synthesis and Prospects

Since the initial discovery implicating miRNAs as essential components of the cardiogenic regulatory network, numerous roles for these small RNAs in cardiac development and disease have been identified. However, of the over 800 miRNAs annotated to-date, only a minute fraction has been studied in the context of the heart. Current bioinformatics algorithms have contributed immensely to our understanding of miRNA function. However, the inability of these models to accurately predict miRNA targets represents a significant challenge that must be addressed. A greater understanding of miRNA mechanisms of action, including the role of non-seed regions, will improve our ability to identify miRNA-mRNA interactions. Moreover, the recent discovery that miRNAs can also function as transcriptional activators may reveal further layers of complexity with respect to the relationships between miRNAs and their targets.

Although we now recognize that miRNAs function in virtually every facet of cell physiology, the field of miRNA biology is only just emerging. Undoubtedly, the expansion of this area of research will uncover novel prospective therapeutic targets for individuals with cardiovascular disease. Further studies seeking to enhance our understanding of the role of miRNAs in cardiogenesis and cardiac regeneration, as well as those designed to ameliorate current experimental and therapeutic methodologies, are required to develop new therapies that impart significant clinical benefits to patients with congenital and acquired cardiac disorders.
